# Regulation as modulation: autonomic flexibility as a physiological buffer in bipolar II disorder — a perspective on somatic regulation and mood stability

**DOI:** 10.3389/fpsyt.2025.1726708

**Published:** 2025-12-17

**Authors:** Kyndra Purcell

**Affiliations:** Independent Researcher, Idaho Falls, ID, United States

**Keywords:** bipolar II disorder, autonomic nervous system, heart-rate variability, vagal tone, somatic regulation, mood stabilization, neurovisceral integration

## Abstract

Bipolar II disorder is traditionally understood as a condition of mood dysregulation, yet beneath its psychological manifestations lies a physiological rhythm often overlooked: the regulation of the autonomic nervous system. This perspective proposes that while bipolar II is not caused by a dysregulated nervous system, its course—and particularly the depth and duration of depressive episodes—is shaped by it. Drawing from psychophysiological literature and lived observation, the paper introduces the concept of regulation as modulation, suggesting that autonomic flexibility functions as a physiological buffer influencing mood stability over time. Heart-rate variability (HRV), vagal tone, and neurovisceral integration are discussed as key biomarkers linking emotional resilience and physiological coherence. When these systems are compromised, recovery from depressive episodes slows; when strengthened through conscious regulation—such as breathwork, interoception, grounding, and somatic awareness—recovery accelerates. The paper integrates evidence from existing studies on HRV and affective regulation with longitudinal self-monitoring data, noting consistent patterns between periods of autonomic dysregulation and the intensity of bipolar lows. This perspective argues that somatic regulation should be regarded as integral to treatment alongside medication and psychotherapy. Rather than framing regulation as auxiliary self-care, it should be viewed as a core therapeutic pathway that restores coherence between body and brain. For clinicians, this approach expands the framework of bipolar care; for researchers, it opens new avenues for investigating physiological mechanisms underlying mood stability. Ultimately, nervous-system regulation is not merely about achieving calm—it is about restoring rhythm, remembering safety, and redefining recovery for those living with bipolar II disorder.

## 1 Introduction — the problem of depth and duration

Bipolar II disorder affects an estimated 1–2 percent of the population, yet its impact extends far beyond diagnostic prevalence. It is often defined less by the height of its hypomanic states than by the *depth and persistence of its lows.* Depressive episodes in bipolar II are typically longer, darker, and more resistant to standard interventions than those seen in unipolar depression—remaining the leading contributor to functional impairment and suicide risk among mood disorders. For many, the illness unfolds not as a dramatic cycle of mania and collapse but as a quieter, chronic instability of energy, emotion, and recovery.

Despite decades of pharmacological and psychotherapeutic progress, full remission remains uncommon. Mood stabilizers, antidepressants, and antipsychotics can reduce frequency of episodes, yet they rarely alter the *physiological terrain* that predisposes instability. Psychotherapies provide valuable insight and behavioral strategies, but even with adherence and awareness, many individuals experience episodes that arise and resolve on their own schedule. The depth and duration of the depressive phase remain the least responsive aspect of treatment—the place where science continues to fall short.

While psychiatric research has long centered on neurotransmitters and cognition, comparatively little attention has been paid to the body’s regulatory systems that underpin emotional resilience. The autonomic nervous system (ANS)—the network coordinating heart rate, respiration, and visceral equilibrium—shapes how stress and emotion are experienced and recovered from. Altered autonomic activity, reflected in reduced heart-rate variability (HRV) and diminished parasympathetic tone, has been documented across depression, anxiety, and trauma-related conditions, yet it is seldom integrated into bipolar research except as a medication side effect or comorbidity marker.

Through extensive review of current literature and reflection on personal experience living with bipolar II, it became clear that the state of the nervous system profoundly influences the intensity of mood descent and the pace of recovery. When the body remains trapped in sympathetic dominance—what many describe as chronic “fight, flight, or freeze”—emotional thresholds narrow and depressive episodes deepen. When the body regains flexibility, able to shift fluidly between activation and rest, mood fluctuations soften and stability lengthens. This pattern, repeatedly echoed in both research findings and lived reports, points toward a neglected dimension of the disorder.

This perspective emerges from that recognition: that bipolar II may not be caused by a dysregulated nervous system, but its course is unmistakably shaped by it. The paper proposes that *autonomic flexibility*—the capacity to move dynamically between arousal and restoration—acts as a *physiological buffer* that can modulate episode depth and duration. By synthesizing existing psychophysiological evidence with a lived-experience lens, this work invites a reframing of treatment focus: from managing symptoms solely in the mind to cultivating regulation in the body as a foundation for mood stability.

## 2 Evidence of autonomic dysregulation in mood disorders

If the previous section named the *problem of depth and duration*, this one looks beneath it—not in metaphor but in physiology. Across decades of psychophysiological research, one pattern keeps resurfacing: the autonomic nervous system (ANS) doesn’t just respond to emotion; it helps shape it.

Heart-rate variability (HRV), the measure of tiny differences between heartbeats, has become one of the clearest windows into this system. Higher HRV reflects a body that can move fluidly between activation and rest; lower HRV reflects rigidity—a system caught in survival mode. Across depression, anxiety, and trauma-related conditions, HRV is reliably reduced, signaling diminished parasympathetic (vagal) tone and chronic sympathetic overdrive (1, 2).

Meta-analyses show that this pattern is cross-diagnostic. People living with depression, post-traumatic stress, schizophrenia, or substance-use disorders all tend to show lower HRV than healthy controls. Within bipolar disorder, the findings echo the same theme: HRV is lowest during depressive or mixed states and only partially recovers in remission, rarely matching non-clinical levels. Some studies even link the degree of HRV suppression to illness duration and symptom intensity—suggesting that the nervous system’s flexibility may not simply mirror mood instability but *modulate* it (3).

Yet most of this research stops at description. HRV is treated as a biomarker to observe, not as a pathway to engage. Few studies ask whether improving autonomic flexibility—through breathwork, vagal stimulation, or somatic awareness—might influence mood stability itself. That absence is what called me to write this paper: to explore the possibility that *regulation is not merely reactive to mood but may be capable of reshaping it*.

Over the past six months, I’ve tracked my own HRV nightly on a Garmin device while living with bipolar II. The numbers have started to tell a story that mirrors the literature. My average HRV sits between 29 and 39 milliseconds, but it consistently drops into the 24–28 range in the days just before and during a depressive low—a pattern I’ve now watched repeat enough times to recognize. When I practice slow, diaphragmatic breathing, gentle somatic stretching, and daily grounding work, my HRV climbs back into the upper 30s. Those are the days that feel most stable—the ones without the heavy pull downward or the quiet thoughts of wanting to disappear. It isn’t a cure, but the correlation is steady: the more regulated my body feels, the steadier my mind becomes.

This longitudinal self-observation, though limited to a single individual, illustrates a principle increasingly supported in the literature: that autonomic regulation influences the experience and trajectory of mood. The body’s rhythm and the mind’s rhythm move together. Understanding that link—and learning how to work with it—is what leads into the next section: the *Modulation Model*, where regulation itself becomes the buffer that softens the amplitude of bipolar lows.

## 3 The modulation model — how regulation buffers the depth and duration of mood episodes

If the autonomic nervous system influences how deeply we descend, then the way we engage it may determine how long we stay there. The *modulation model* proposes that the degree of physiological regulation acts as a buffer—shaping not only the *intensity* of bipolar lows but also their *duration* and *recovery curve*. In this view, mood is not isolated in the brain but expressed through a rhythm dialogue between the mind and body. When that rhythm loses coherence, lows deepen: when coherence is restored, the body becomes an anchor through which the mind can return.

Through daily practice, I’ve learned that regulation does not erase depression. Episodes and intrusive thoughts still arise, but with less intensity and shorter duration. They still visit—but the difference is in their power. When I remain consistently regulated, those waves arrive gentler, less catastrophic, and easier to ride. The storm still forms, but it no longer sweeps me under. Conscious regulation—breathing, stretching, grounding, and attuning to bodily signals—doesn’t silence the illness, but it changes the weather around it.

Over time, a clear pattern emerges; when external demands intensify—through stress, illness, or collective unrest—the coherence of my nervous system wavers. If I skip the practices that keep me anchored, *the lows last longer and cut deeper.* Episodes that once passed in a week stretch into three. The fog thickens; the return to baseline drags. My HRV drops into the 20s until I rebuild coherence through breath and movement. Each time, the pattern repeats: neglecting regulation extends suffering; tending to it shortens the storm.

This is the essence of *regulation as modulation*. The goal is not to cure bipolar disorder or eliminate its cycles, but to reshape their amplitude and rhythm. Autonomic regulation becomes a way to influence the physiological terrain on which mood episodes unfold. Just as the ocean’s tides are governed by the moon’s pull, mood states seem governed by the body’s capacity to stabilize and recover from stress. In this light, the work of regulation is not self-care in the casual sense, but a *form of physiological stewardship*—an ongoing dialogue with the nervous system that determines how rough the waters become and how quickly calm can return. From a scientific standpoint, this aligns with the *allostatic-load* model: the cumulative strain of stress weakens resilience, while daily regulation practices restore it. It also reflects the *neurovisceral-integration* framework, where vagal flexibility supports adaptive emotional control. From a lived standpoint: the more regulated my body is, the more survivable my mind becomes.

## 4 Mechanisms — how body and brain communicate in mood regulation

Mood and physiology are not separate systems—they are interwoven loops of feedback, continually shaping one another. Feeling is, in part, the brain interpreting the language of the body. When that language becomes chaotic or incoherent, so does emotion.

At the center of this communication is the autonomic nervous system—specifically, the dialogue between its sympathetic and the parasympathetic branches. The sympathetic system mobilizes energy for stress; the parasympathetic system, mediated largely by the vagus nerve, restores calm, digestion, and repair. Mental health depends not on the absence of either branch but on their ability to dance together—to activate, release, and return to balance. This capacity, called *autonomic flexibility*, is the physiological basis of resilience (4).

When flexibility is lost, as seen in chronic stress or trauma, the body “lives” in the sympathetic branch. Heart rate increases, breathing shallows, digestion slows, cortisol rises. Over time, this defensive posture becomes the emotional landscape itself—irritability, fatigue, hopelessness, and the sense that joy demands effort. In bipolar II, this loss of flexibility becomes a silent amplifier; not the cause of the low, but a magnifier of its depth and duration.

My own nervous system mirrors this. When I overextend—skip rest, or absorb collective tension—breathing shallows, HRV falls, sleep loses depth, and mood darkens. Within days, my thoughts and emotions begin to echo the state of my body. When I return to regulation through breathwork and grounding, HRV climbs and a different kind of conversation begins: one where the body signals safety, and the mind can finally listen.

The *neurovisceral-integration model* helps explain why. It proposes that HRV reflects the connectivity between the prefrontal cortex—responsible for self-regulation and decision-making—and the limbic system, which governs emotion. When HRV is high, these regions communicate fluidly; when low, the connection weakens, and emotion overrides reasoning (5). A calm body supports coherent thought; a tense body fragments it.

Recent extensions of Thayer and Lane’s framework clarify how prefrontal-vagal pathways regulate emotional balance through a continuous, two-way dialogue between the brain and body. Neuroimaging studies show that individuals with higher vagally mediated HRV exhibit stronger coupling between the ventromedial prefrontal cortex and the amygdala during emotion-regulation tasks (6, 7). This synchrony allows the body to modulate emotional intensity rather than be overwhelmed by it—a physiological conversation that underlies psychological resilience. Reduced vagal tone predicts weaker top-down regulation and heightened emotional volatility—features observed in affective disorders including bipolar illness (8, 9). These findings reinforce HRV as a mirror of functional connectivity—an index of how coherently the body and brain communicate during affective challenge.

From this lens, autonomic rhythm becomes a real-time expression of integration. The prefrontal cortex may attempt to think its way to safety, but lasting regulation arises when the body itself signals calm and coherence. Healing, then, happens through this partnership: the brain learns stability through the steady signals the body sends it. Regulation is not about suppressing emotion—it is about reestablishing rhythm so that both systems remember how to communicate again.

The *allostatic-load model* deepens this view. It describes how accumulated stress gradually taxes the body’s adaptive systems, reducing the ability to recover (10). Each unregulated stressor—sleep deprivation, illness, emotional strain, even collective tension—adds to the body’s load. Without consistent down-regulation, that load manifests as both physical and emotional exhaustion. The longer the nervous system remains dysregulated, the longer the depressive recovery curve. This aligns closely with my own observations: when regulation falters for days or weeks, depressive lows extend and recovery slows; when regulation is steady, the return to baseline accelerates.

In essence, the communication between body and brain is the foundation of modulation itself. Regulation does not force happiness; it restores signal coherence—a physiological harmony that allows the mind to process emotion without being consumed by it. Each breath, each movement, each moment of awareness sends a message through the vagus nerve, telling the body it is safe to soften. Repeated over time, those messages become the rhythm that steadies both physiology and mood. This is the hidden mechanism behind stability: the brain does not heal in isolation—it heals through the steady signals of the body, through the quiet daily choices that rewire chaos into calm. And in bipolar II—where the mind can feel like a storm too vast to reason with—it is often the body that provides the first glimpse of a way out.

## 5 Somatic regulation as an intervention — reclaiming the body as a tool for stability

If autonomic flexibility is the foundation of resilience, then regulation is the practice that builds it. The nervous system cannot be reasoned with in language—it must be spoken to through sensation, rhythm, and breath. This is why somatic regulation becomes so essential in bipolar II and other mood disorders: it gives the body a vocabulary to calm itself when the mind cannot. Regulation is not about controlling emotion; it is about *creating the physiological conditions in which emotion can move through*. Each time the body returns to safety, even briefly, it sends a message through the vagus nerve to the brain: *you can stand down.* Over time, that message rewires the feedback loop between body and mind, transforming survival into steadiness.

For me, regulation has become less a technique and more a practice of reconnection. I begin most mornings with breathing work—long exhales that anchor me into my body. Some days I include somatic movements to release the shoulders and hips where tension gathers. At night, I take hot showers, not for comfort alone but for the way heat softens my muscles and slows my pulse and quiets the mind. I ground by stepping barefoot onto the earth, letting the cold or the grass remind me that the world is still here. I spend time in nature—listening, walking, and attuning to the elements that mirror the body’s own rhythm and capacity for regulation. These practices don’t erase depression. The lows still come, but they move through a body that remembers how to return to balance.

A growing body of research now supports the physiological basis of these lived experiences. Exercise interventions reliably enhance parasympathetic tone and improve emotion regulation (11). HRV-biofeedback and slow-breathing protocols increase vagally mediated HRV and reduce depressive symptoms (12, 13). Mindfulness and meditation practices strengthen vagal regulation through attentional control and interoceptive awareness (14). Even nutritional and lifestyle factors, such as omega-3 fatty-acid supplementation, have been linked to improved autonomic balance (15).

These converging findings demonstrate that practices like breathwork, movement, and grounding share common physiological mechanisms: they signal safety through the vagus nerve and recalibrate autonomic rhythm. The lived sense of regulation therefore aligns with measurable neurocardiac change—a reminder that self-care is not anecdotal but biologically plausible. From a physiological perspective, these practices stimulate the parasympathetic branch of the ANS, increasing vagal tone and HRV. From a lived perspective, they restore authorship—something to *do* when the mind feels helpless. They become a bridge between biology and meaning: a way of saying to the body, *You are safe now. You can rest.* This is why somatic regulation deserves recognition not as an adjunct to therapy but as a core component of mood stabilization.

## 6 Implications for research and clinical practice — reframing stability as regulation

If the nervous system shapes how deeply and how long a person experiences a mood episode, then regulation should not remain an afterthought to treatment—it should stand beside medication as a central part of care. Bipolar II is not cured by pharmaceuticals alone. While medications stabilize neurochemistry, it does not always restore coherence to the body, it may quiet the noise, but it cannot retrain the rhythm.

This paper does not suggest that nervous-system regulation replace medication. Rather, it proposes that conscious engagement with physiology is as essential to long-term stability as pharmacological management. When the nervous system is steady, the storms of bipolar II still occur, but they no longer decide the self within them.

Clinical, this reveals a gap that could be filled by teaching intentional regulation. Breathing exercises, grounding, body-based mindfulness, and time in nature are not wellness trends; they are physiological interventions that rebalance a system designed for survival. Even simple actions——slow diaphragmatic breathing, gentle stretching of the intercostal muscles, or humming softly to stimulate the vagus nerve—can recalibrate the system. Patients do not need identical methods; what matters is the *effect*: anything that reduces arousal and increases coherence functions as regulation. If clinicians prescribe these practices with the same seriousness as medication—if daily regulation became a formal part of care—the outcomes could be profound. Patients would not only have something to *take* but something to *do*—a skill accessible even when the mind feels unreachable.

For researchers, the path forward is clear. Future studies could examine how consistent autonomic regulation impacts HRV, relapse rates, and recovery time in bipolar populations. Randomized trials pairing standard medication with somatic training could test whether regulation reduces the duration or severity of depressive episodes. The integration of wearable HRV technology offers a way to quantify physiological progress alongside psychological outcomes, bridging subjective experience with measurable data. Ultimately, this perspective reframes treatment as a partnership between the medical and the embodied. Medication supports chemistry; regulation supports rhythm. The first stabilizes what the brain produces; the second stabilizes how the body receives it. Together they create a foundation for real resilience—a healing that honors the full truth of bipolar disorder: that it lives in both mind and body, and both deserve equal care.

## 7 Conclusion — regulation as remembering

Bipolar II is often described as a disorder of mood, but beneath that definition lies something quieter: a disorder of rhythm. The body forgets how to return to safety, and the mind forgets what safety feels like. The work of regulation, then, is, not management—it is remembrance.

This perspective has argued that bipolar II is not caused by a dysregulated nervous system, but its course is undeniably shaped by it. The deeper the dysregulation, the deeper the lows; the steadier the regulation, the steadier the return. Through both research and lived observation, it becomes clear that *autonomic flexibility is a physiological buffer*—a mediator that transforms how individuals experience and recover from their episodes.

Medication can balance chemistry; therapy can reframe thought. But *regulation restores rhythm*—it reintroduces the body to the feeling of safety that medication alone cannot teach. For those living with bipolar II, this realization can become a kind of freedom: knowing that while we may not control the storm, we can strengthen the vessel that carries us through it.

Stability ceases to be a static goal and becomes an ongoing relationship with the body’s wisdom. For clinicians, this is an invitation to treat regulation not as optional, but essential. For researchers, it opens the door to exploring new pathways between physiology and emotion. And for anyone living with bipolar disorder, it offers something deeply human: the understanding that healing may begin not in the mind, but in the breath, the heartbeat, and the body’s gradual return to safety. In the end, regulation is not about calm—it is about coherence. It is the act of remembering that the body was never the enemy, only the messenger. When we learn to listen, we discover that the path to stability has always been rhythmic, intimate, and within reach.

## Author’s note

This perspective is written from a lived-experience standpoint, integrating self-observation with a review of current research on autonomic regulation and mood disorders. The author conceptualized, drafted, and refined this manuscript in full. Foundational theories and empirical research on autonomic regulation, vagal tone, and allostatic load are credited to prior work by Thayer & Lane (5) Beauchaine & Thayer (8), McEwen (1998), Wendt & Thayer (15) Kemp & Quintana (16), and Moss (17). The *Modulation Model™* and its framing of “regulation as modulation” are original contributions integrating existing psychophysiological evidence with a new perspective on mood trajectory and recovery in bipolar II disorder.

## Data Availability

The original contributions presented in the study are included in the article/supplementary material. Further inquiries can be directed to the corresponding author.
